# Occurrence of circumscribed choroidal hemangioma accompanied by polypoidal choroidal vasculopathy and branch retinal vein occlusion: a case report

**DOI:** 10.1186/s12886-023-02880-x

**Published:** 2023-04-26

**Authors:** Qin Zhang, Huan Wang, Yue Zhang, Ling Xu

**Affiliations:** Shenyang He Eye Specialist Hospital, No. 128 Huanghe North Street, Shenyang, 110034 Liaoning Province China

**Keywords:** Circumscribed choroidal hemangioma, Polypoidal choroidal vasculopathy, Hypertension, Branch retinal vein occlusion

## Abstract

**Background:**

Circumscribed Choroidal hemangioma (CCH) is a kind of hamartoma that is caused by congenital vascular malformation. And, polypoidal choroidal vasculopathy (PCV) is an exudative maculopathy. There is no literature indicating that there is a correlation between the occurrences of CCH and PCV.

**Case presentation:**

A 66-year-old male presented with decreased vision of his left eye for 4 years. Fundus photograph showed that the branches of blood vessels at the supratemporal retina were occluded in white lines, an orange lesion could be seen in the subnasal retina and mottled, yellowish white lesions were accompanied by punctate hard exudation in the macular in the left eye. The Fundus autofluorescence (FAF), fundus fluorescein angiography (FFA), indocyanine green angiography (ICGA) and Spectral domain optical coherence tomography (OCT) were done. There was a diagnosis of CCH, PCV and branch retinal vein occlusion accompanied with retinoschisis of the left eye.

**Conclusion:**

This article reports on a case of an elderly male Chinese patient with CCH and PCV accompanied by branch retinal vein occlusion with retinoschisis in the left eye. The common lesions are choroidal vascular abnormalities. Whether hypertension is related to CCH, PCV and branch retinal vein occlusion remains to be further studied.

## Background

Choroidal hemangioma is a kind of hamartoma that is caused by congenital vascular malformation, which can be divided into diffuse and circumscribed types [1]. Patients with facial and cutaneous hemangiomas are said to have Sturge-Weber syndrome [2]. Circumscribed choroidal hemangioma (CCH) is more common, and mainly occupies the choroidal vascular layer [1]. The CCH is usually asymptomatic and often diagnosed in adulthood during a routine eye examination [1].

Polypoidal choroidal vasculopathy (PCV) is an exudative maculopathy characterised by multiple recurrent serosanguineous retinal pigment epithelial detachments [3]. Yannuzzi first reported on this in1982 at a meeting of ophthalmology Conference [3]. Polypoidal choroidal vasculopathy mostly presents in black women, Asian men and people over 60 years old [4]. The cause of PCV is unclear, but it may be related to age and race [4]. Although the occurrence of CCH and PCV is related to choroidal abnormalities, there is no literature indicating that there is a correlation between the occurrences of each.

This article reports on a case of an elderly male Chinese patient with CCH and PCV accompanied by branch retinal vein occlusion with retinoschisis in the left eye.

## Case presentation

**Fig. 1:**
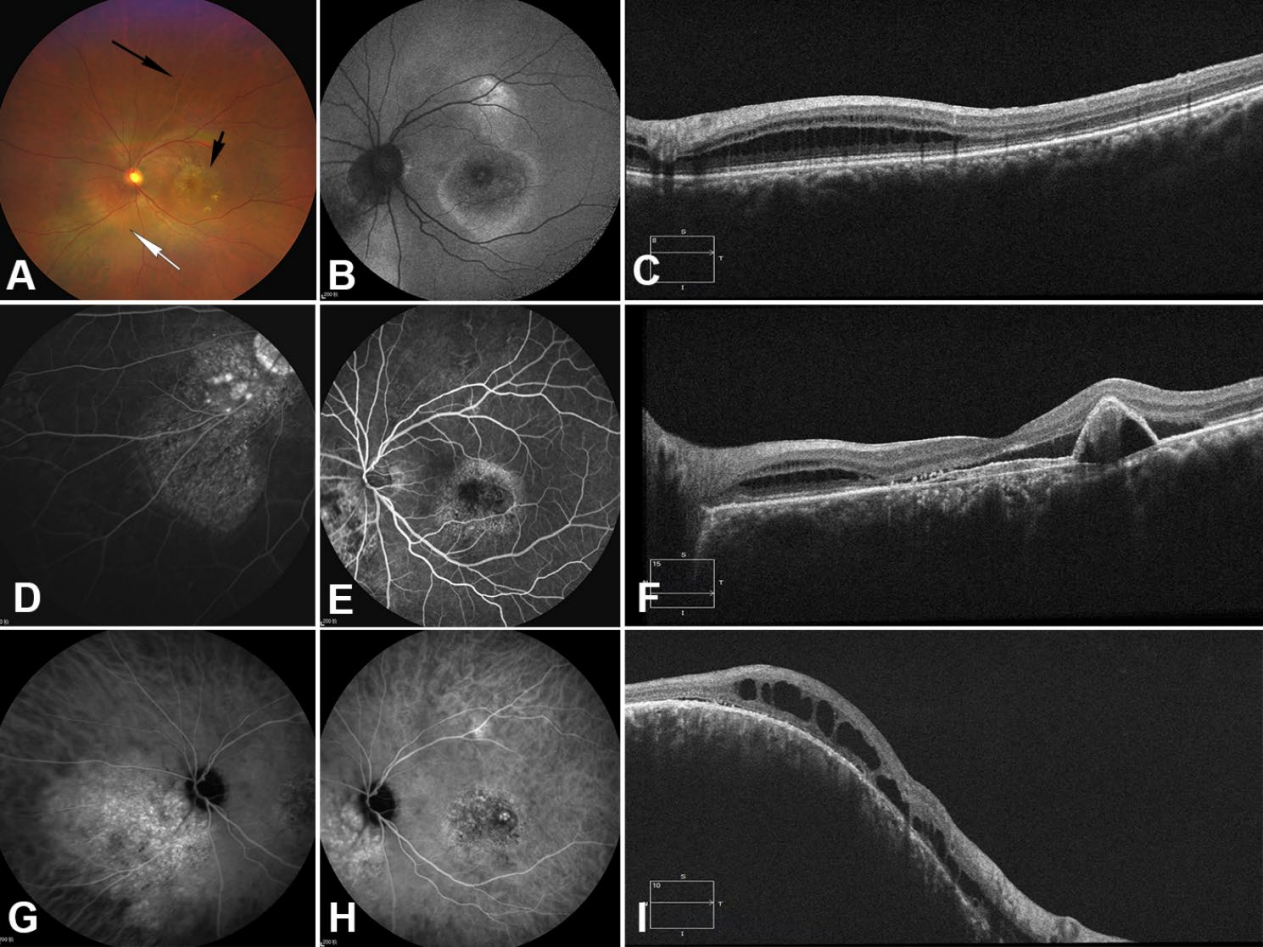
Photographs of the fundus in the patient’s left eye (A-I). (A) Ophthalmoscopy revealed an orange lesion in the subnasal retina and mottled yellowish white lesions accompanied by punctate hard exudation in the macular. (B) Fundus autofluorescence showed disappeared branches of blood vessels at the supratemporal retina, a round weak fluorescence in the subnasal retina and a ringlike hyperfluorescence in the macular. (C) Optical coherence tomography revealed vertical bridging tissues in the inner layer of the supratemporal retina. (D) Fundus fluorescein angiography showed a large calabash-shaped hyperfluorescence lesion in the subnasal retina. (E) Fundus fluorescein angiography showed the occluded blood vessel at the supratemporal retina and mottled high fluorescence lesions in the macular. (F) Optical coherence tomography revealed a double-layer sign and sharp-peaked pigment epithelial detachment in the macular. (G) Indocyanine green angiography (ICGA) showed a large area, high fluorescence lesion in the subnasal choroid. (H) Indocyanine green angiography showed mottled high fluorescence with a cluster of polyps in the macular. (I) Optical coherence tomography revealed choroidal upwelling accompanied by inner retina oedema at the inferior retina

A 66-year-old male presented with decreased vision accompanied by metamorphopsia of his left eye for 4 years. During the 4 years, he didn’t see a doctor and had not received any treatment. He had no systemic diseases, such as diabetes or heart disease. His blood pressure was 140/110 mmHg. The best corrected vision (decimal) was 0.8 in his right eye and finger counting in his left eye. The intraocular pressure was within normal limits in both eyes. There was light opacity in the bilateral lens. A fundus examination (Zeiss, Germany) showed that the branches of blood vessels at the supratemporal retina were occluded in white lines, an orange lesion could be seen in the subnasal retina and mottled, yellowish white lesions were accompanied by punctate hard exudation in the macular in the left eye (Fig. 1A). There were scattered yellowish white lesions in the macular in the right eye. Fundus autofluorescence (FAF) (Heidelberg Engineering, Heidelberg, Germany) showed branches of blood vessels at the supratemporal retina had disappeared, and there was a round, weak fluorescence in the subnasal retina and ringlike hyperfluorescence in the macular in the left eye (Fig. 1B). Fundus fluorescein angiography (FFA) (Heidelberg Engineering, Heidelberg, Germany) showed that branches of blood vessels at the supratemporal retina were occluded, and there was a large calabash-shaped hyperfluorescence lesion in the subnasal retina and mottled high fluorescence lesions in the macular in the left eye (Fig. 1D, E). There was mottled high fluorescence lesions in the macular without leakage in the right eye. Indocyanine green angiography (ICGA) (Heidelberg Engineering, Heidelberg, Germany) showed fluorescein staining in hemangioma at infranasal retina of the early stage with high fluorescence of lesions at intermediate stage (Fig. 1G) and fluorescein washed out at the late stage in the left eye. ICGA also revealed mottled high fluorescence with a cluster of polyps in the macular (Fig. 1H). There was no branching vascular network (BVN) was found. There were mottled high fluorescence lesions, which vanished gradually at the late stage in the macular in the right eye.

Spectral domain optical coherence tomography (SD-OCT) (Heidelberg Engineering, Heidelberg, Germany) revealed a double-layer sign and sharp-peaked pigment epithelial detachment in the macular (Fig. 1F), as well as choroidal upwelling accompanied by inner retina oedema at the inferior retina (Fig. 1I). Vertical bridging tissues were seen in the inner layer of the supratemporal retina (Fig. 1C) in the left eye. Retinal pigment epithelial in the macular of the right eye is disordered. The colour Doppler blood flow imaging (CDFI) revealed that a prominent lesion with clear boundary and blood flow signal can be seen on the wall of the posterior pole, whose size was about 0.92 cm*2.28 cm. Laboratory examinations were normal, including tests for syphilis antibody, human immunodeficiency virus and hepatitis. According to the clinical characteristics and examination results, there was a diagnosis of CCH, PCV and branch retinal vein occlusion accompanied with retinoschisis of the left eye. The diagnosis was dry, age-related macular degeneration of the right eye. The patient refused to do laser photocoagulation because of the possible risks. He received intravitreal injection of anti-VEGF drugs twice but there was not much effect.

## Discussion and conclusions

The CCH is a benign vascular tumour that is not accompanied by facial or systemic diseases [2]. The main symptoms of CCH are decreased vision and visual distortion [2]. The fundus is characterised by extensive, flat and borderless tomato colour thickening at the posterior pole, which may be accompanied by exudative retinal detachment or cystic degeneration [1]. The cause of CCH was believed to be related to congenital vascular malformation [1]. In histopathology, the growth and expansion of CCH is more likely due to the vasodilation of the choroidal vessels than cell proliferation [5]. In recent years, cases of CCH have been reported in the literature and CCH was no longer rare. In 2015, Morgan Berry reported a 49-year-old man with a CCH superior to the optic nerve in the left eye [6]. In 2022, Vijitha S. et al. described a 32-year-old Indian woman with multifocal CCH in her left eye, which was rare [7]. In 2018, Rayan S. Kim et al. found that the elevated choroid thickness was related to a risk of developing CCH, as well as central serous chorioretinopathy in patients of varying ages, and proposed that patients diagnosed with CCH should be screened for central serous chorioretinopathy in the fellow eye [8].

What makes the patient in this article special is that he had CCH, but also had PCV and branch retinal vein occlusion simultaneously. It is unknown whether there is a relationship between the occurrence of CCH and PCV. Polypoidal choroidal vasculopathy was similar to neovascular, age-related macular degeneration in morphological characteristics, [3] which was common and often reported in the literature. It was believed to be caused by inner choroidal vessel abnormalities [3]. Thinning of the inner wall of the choroidal blood vessel leads to the formation of multiple small polypoid aneurysms. [9] Currently, hypertension, raised plasma viscosity and thrombocytopenia are considered related to PCV, [9] but it is still considered debatable. In addition, Dalvin et al. found that 39 out of 94 patients with CCH in the age group older than 50 years old had hypertension, and 14 out of 25 patients with CCH in the age group from 20 to 50 years old [10]. Hypertension may be relevant with branch retinal vein occlusion and PCV. Therefore, whether it can be inferred that hypertension is the disease basis of the simultaneous occurrence of CCH, PCV and branch retinal vein occlusion. It may be that hypertension causes choroidal hypertension, which leads to choroidal damage, thus causing a series of choroidal lesions. We proposed this mechanism as a hypothesis, which needs a lot of research in the future.

Furthermore, the patient suffered from retinoschisis at the lesion of branch retinal vein occlusion. The mechanism may be that the longer course of branch retinal vein occlusion leads to retinal capillary blood supply disorder, which causes retinal cell apoptosis and inner retinal cystic degeneration. Then, the cortex covering the vitreous body shrinks, causing traction of the inner retina, and finally forming retinoschisis.

The therapy of simple CCH contains laser photocoagulation and PDT. This patient refused to do laser photocoagulation because of the possible risks. And we did not have the PDT treatment at present. The therapy of our case should contain intravitreal injection of anti-VEGF drugs or PDT for PCV and laser photocoagulation for BRVO or CCH. However, the patient only received intravitreal injection of anti-VEGF drugs twice and there was not much effect. He gave up the treatment at last.

In conclusion, this article is the first report of a rare case of simultaneous CCH, PCV and branch retinal vein occlusion, as far as we know. The common lesions are choroidal vascular abnormalities. Whether hypertension is related to CCH, PCV and branch retinal vein occlusion remains to be further studied.

## Data Availability

All data generated or analysed during this case are included in this published article.

## Abbreviations

CCH: Circumscribed Choroidal hemangioma
PCV: polypoidal choroidal vasculopathy
FAF: Fundus autofluorescence
FFA: Fundus fluorescein angiography
ICGA: Indocyanine green angiography
SD-OCT: Spectral domain optical coherence tomography
